# The Heme Transporter HtsABC of Group A *Streptococcus* Contributes to Virulence and Innate Immune Evasion in Murine Skin Infections

**DOI:** 10.3389/fmicb.2018.01105

**Published:** 2018-05-25

**Authors:** Yingli Song, Xiaolan Zhang, Minghui Cai, Chunmei Lv, Yuan Zhao, Deqin Wei, Hui Zhu

**Affiliations:** Department of Physiology, Harbin Medical University, Harbin, China

**Keywords:** group A *Streptococcus*, *Streptococcal* heme transporter A (HtsA), virulence, host immune response, phagocytosis, cytokines

## Abstract

Group A *Streptococcus* (GAS) requires iron for growth, and heme is an important source of iron for GAS. Streptococcus heme transporter A (HtsA) is the lipoprotein component of the GAS heme-specific ABC transporter (HtsABC). The objective of this study is to examine the contribution of HtsABC to virulence and host interaction of hypervirulent M1T1 GAS using an isogenic *htsA* deletion mutant (Δ*htsA*). The *htsA* deletion exhibited a significantly increased survival rate, reduced skin lesion size, and reduced systemic GAS dissemination in comparison to the wild type strain. The *htsA* deletion also decreased the GAS adhesion rate to Hep-2 cells, the survival in human blood and rat neutrophils, and increased the production of cytokine IL-1β, IL-6, and TNF-α levels in air pouch exudate of a mouse model of subcutaneous infection. Complementation of Δ*htsA* restored the wild type phenotype. These findings support that the *htsA* gene is required for GAS virulence and that the *htsA* deletion augments host innate immune responses.

## Introduction

*Streptococcus pyogenes* or Group A *Streptococcus* (GAS) is a major Gram-positive pathogen that causes a variety of human diseases. The most common GAS infections are mild, including pharyngeal infections like pharyngitis and skin, soft tissue infections like impetigo and erysipelas. Occasionally, GAS can cause severe invasive infections with high morbidity and mortality, such as necrotizing fasciitis, bacteremia, and toxic shock syndrome (Henningham et al., 2012; Bessen et al., 2015) or postinfection sequelae such as acute poststreptococcal glomerulonephritis (APSGN), acute rheumatic fever (ARF), and rheumatic heart disease (RHD) (Cunningham, 2008; Zühlke et al., 2017).

Iron is an essential nutrient for the growth and survival of most bacterial pathogens (Sheldon et al., 2016). Heme is the major iron source for bacterial pathogens in mammalian hosts (Eichenbaum et al., 1996; Rouault, 2004). *Streptococcal* hemoprotein receptor (Shr), *streptococcal* heme binding protein (Shp) and *streptococcal* heme-specific ABC transporter (HtsABC) encode a machine that takes up and transports heme in GAS. In the heme acquisition pathway (Lei et al., 2002; Bates et al., 2003; Zhu et al., 2008), Shr obtains heme from heme-containing proteins such as hemoglobin and myoglobin and efficiently transports it combined with heme to Shp. Heme transporter A (HtsA) protein is a lipoprotein component of HtsABC that obtains heme from Shp as an iron source for GAS survival (Nygaard et al., 2006; Zhu et al., 2008; Lu et al., 2012). The *shr*, *shp*, and *htsABC* genes are located in the same operon, which is regulated by MtsR, a transcriptional regulator that can directly regulate the transcription of some genes related to heme and metal ion uptake (Hanks et al., 2006).

Some studies have reported that iron and heme uptake proteins are associated with the virulence and pathogenicity of bacteria (Palmer and Skaar, 2016) such as *Streptococcus pneumonia* (Brown et al., 2001), *Staphylococcus aureus* (Skaar et al., 2004), *Vibrio cholera* (Mey et al., 2005), enterotoxigenic *Escherichia coli* (Haines et al., 2015), and *Pseudomonas aeruginosa* (Minandri et al., 2016). In GAS, few publications have investigated the relationship between iron and heme uptake proteins and the virulence of GAS. Our laboratory recently reported that another heme uptake protein, Shp, is important for GAS virulence (Zhang et al., 2017). However, whether HtsA is also important for the virulence of GAS is unknown. In this study, we investigated the contribution of HtsA to GAS virulence.

## Materials and Methods

### Bacterial Strains, Media, and Growth Conditions

MGAS5005 (serotype M1), a well-characterized hypervirulent M1T1 GAS isolate (Nasser et al., 2014), was used as the parent strain for the construction of an *htsA* deletion mutant in this study. For routine growth, GAS strains were grown at 37°C in 5% CO_2_ in Todd-Hewitt broth (Difco Laboratories) supplemented with 0.2% yeast extract (THY).

### Construction of the *htsA* Deletion Mutant Strain (Δ*htsA*) and Revertant Strain (Δ*htsA-htsA*)

An in-frame deletion mutant of *htsA* lacking an internal fragment from amino acids Asp152 to Phe252 was constructed as previously described (Zhu et al., 2009). Briefly, a 978-bp upstream fragment containing the first 453 nucleotides (amino acids 1-151) of the *htsA* gene (GenBank Gene ID NC-002737) and a 1,129-bp downstream fragment containing the last 129 nucleotides (253-295 amino acids) of the *htsA* gene were amplified from the MGAS5005 chromosome with the following primers: upstream fragment, CGAGATCTgacaacactagatattgctattag (BglII site) and GTCTCGAGctggtattgctggcgcaattct (XhoI site); downstream fragment, AGCTCGAGacggcagtcaaggaagggaaag (XhoI site) and CTGGATCCggtgcttacttccaaccagcaag (BamHI site) (primer sequences are given left to right in 5′-to-3′ order, underlined sequences represent the restriction endonuclease sites). The PCR-derived fragments were then sequentially cloned into the suicide plasmid pGRV. The plasmid was introduced into MGAS5005 by electroporation to generate the *htsA* mutant strain designated Δ*htsA* by two-step crossover homologous recombination. The revertant strain designated Δ*htsA-htsA* was constructed using the same two-step homologous recombination strategy. Briefly, a 2000-bp PCR fragment containing the 885-bp wild type full-length *htsA* gene and its flanking regions was cloned into pGRV. The resulting plasmid pGRV-*htsA* was introduced by electroporation into the Δ*htsA* mutant strain to construct a revertant strain. The mutant and revertant were verified by PCR analysis and further confirmed by DNA sequencing to rule out spurious mutations.

### Detection of HtsA by Western Blotting Analysis

HtsA protein expression in GAS strains was detected by western blotting analysis. To prepare samples for the analysis, bacteria were harvested at the exponential growth phase, washed twice with PBS, and treated with 200 units of mutanolysin in 100 μl of PBS at 37°C for 2 h. The samples were briefly sonicated and mixed with an equal volume of 2× SDS-PAGE loading buffer. Proteins in the samples were separated by SDS-PAGE and transferred from the gel to nitrocellulose membranes. The primary antibody used was an affinity-purified polyclonal rabbit IgG antibody at a dilution 1:500 (antibodies against HtsA and Shp proteins were kind gifts from Dr. Lei at Montana State University). The secondary antibody was a goat antirabbit immunoglobulin G (IgG)-peroxidase conjugate (Bio-Rad Laboratories). Signal detection was conducted with the SuperSignal West Pico chemiluminescent substrate reagent (Pierce Biotechnology).

### Ethics Statement

All animal procedures were approved by the Institute’s Ethics Committee of Harbin Medical University (HMUIRB20170031). All animal experiments were carried out in strict accordance with the regulations in the Guide for the Care and Use of Laboratory Animals issued by the Ministry of Science and Technology of the People’s Republic of China. Animals were euthanized by diethyl ether inhalation.

### Mouse Infection

Female CD-1 mice (5–6 weeks old) obtained from the Animal Center of Heilongjiang Chinese Medicine University were utilized for the invasive subcutaneous model of GAS infection. GAS strains were grown in THY medium to the exponential phase (OD600≈0.6) at 37°C, harvested by centrifugation, washed twice with ice-cold PBS, and adjusted to ∼1.0 × 10^9^ CFU/ml with PBS. To monitor survival rates, groups of mice (*n* = 18) were subcutaneously inoculated with 0.2 ml of ∼2.0 × 10^8^ CFU GAS suspension and observed daily for 15 days. To determine the skin lesion size and the bacterial systemic dissemination from skin into blood and organs, groups of mice (*n* = 7) were subcutaneously inoculated with 0.2 ml of ∼2.0 × 10^8^ CFU of GAS. At 24 h postinoculation, skin lesion sizes were calculated by measuring the length and width at the longest point of the lesion (length by width). The organs, including skin, spleen, liver, lung, and kidney, were weighed and homogenized in PBS using a Kontes pestle. The heparinized blood and tissue homogenates were then plated on THY agar plates in serial 10-fold dilutions and cultured overnight at 37°C to determine the number of viable GAS CFU.

### Adherence and Invasion Assays

Adherence and invasion assays were performed using human laryngeal epithelial (Hep-2) cells as described previously (Timmer et al., 2006). Hep-2 cells were seeded in 24-well culture plates at 2 × 10^5^ cells/well in RPMI 1640 supplemented with 2% FBS and grown to confluence at 37°C with 5% CO_2_. GAS strains were grown to an OD600≈0.6, harvested, and diluted in PBS. Confluent monolayers were washed with PBS and infected with bacteria at a multiplicity of infection (MOI) of 5:1. Plates were centrifuged for 5 min at 800 ×*g* to initiate bacterial contact and incubated for 2 h at 37°C in 5% CO_2_. For adherence assays, cells were washed three times with PBS to remove non-adherent bacteria. For invasion assays, cells were treated with 100 μg/ml gentamicin and 5 μg/ml ampicillin for an additional 2 h to kill extracellular bacteria. After incubation, the cells were washed three times with PBS and lysed with a mixture containing 0.25% trypsin and 0.025% Triton X-100. The lysates were serially diluted, plated on THY agar and incubated overnight at 37°C for enumeration of CFU. Bacterial adherence and invasion were expressed as the adhesion rate or invasion rate: (CFU of adherent or invaded bacteria/CFU of initial inoculated bacteria) × 100. Each assay was performed in triplicate, and all assays were repeated three times.

### GAS Growth in Whole Human Blood

GAS growth in whole human blood was carried out as described previously (Dahesh et al., 2012). Whole human blood was collected from four healthy volunteers in accordance with a protocol approved by the Institute’s Ethics Committee of Harbin Medical University. GAS strains were harvested at exponential phase (OD600≈0.6) and were washed three times with PBS. Finally, 20 μl of ∼2 × 10^4^ CFU bacterial suspension was mixed with 0.5 ml of heparinized blood in siliconized tubes. The blood–bacteria mixtures were incubated at 37°C with orbital rotation and then serially diluted in PBS and plated onto THY agar at time points of 0 min, 30 min, 60 min, 120 min, and 180 min of incubation. Bacterial survival in human blood was estimated from the ratio of the resulting CFU in each sample after incubation to the starting CFU in the inoculum.

### GAS Growth in Rat Neutrophils

Isolated rat neutrophils were purified using HISTOPAQUE-1083 (Siemsen et al., 2007). A GAS suspension was mixed with 1.0 ml of 2 × 10^4^ neutrophils at a MOI of 10:1. The mixture was incubated at 37°C with gentle agitation on a rotating mixer, and 50 μl of the mixture was plated onto THY agar at 0 min, 30 min, 60 min, 120 min, and 180 min of incubation. Bacterial survival in rat neutrophils was estimated from the ratio of the resulting CFU in each sample after incubation to the starting CFU in the inoculum.

### MPO Activity of Neutrophils

Skin lesions of mice containing the infection area were excised and homogenized at 24 h postinfection. The numbers of recruited neutrophils in the excised skin were estimated by the myeloperoxidase (MPO) assay, as described previously (Liu et al., 2012). The MPO activity of neutrophils in the infection area was determined using an MPO detection kit (Nanjing Jiancheng Bioengineering Institute, China).

### Gram Staining

Skin lesions of mice were excised at 24 h postinfection, fixed in 10% buffered formalin, and embedded in paraffin. The paraffin blocks were sectioned at a thickness of 5 μm using a rotary microtome. The sections were stained with 1% crystal violet and Gram’s iodine and then counterstained with 1% basic fuchsin according to standard Gram staining procedure.

### Determination of Cytokines by Enzyme-Linked Immunosorbent Assay (ELISA)

The air pouch model of infection was previously described (Lu et al., 2013). Briefly, groups of mice (*n* = 7) were subcutaneously infected with 2 ml of air to form an air pouch and were inoculated with 0.2 ml of ∼2 × 10^8^ CFU bacterial suspension. At 24 h postinfection, air pouch exudate was collected by injecting 1 ml of PBS into the air pouch and aspirating the exudate. The levels of TNF-α, IL-1β, and IL-6 cytokines in the exudate were determined by ELISA Kit (eBioscience, San Diego, CA, United States) according to the manufacturer’s instructions. The absorbance at 450 nm for each sample was analyzed by an ELISA reader.

### Statistical Analyses

All statistical analyses were performed using GraphPad Prism version 5.0 (GraphPad Software Inc.). The survival data of the mice were analyzed using the log-rank (Mantel–Cox) test. Other data in this study were analyzed by one-way analysis of variance (ANOVA) or two-way ANOVA with the Newman–Keuls multiple-comparison posttest.

## Results

### Construction of the *htsA* Deletion Mutant (Δ*htsA*) and Revertant of Δ*htsA* (Δ*htsA-htsA*)

**FIGURE 1:**
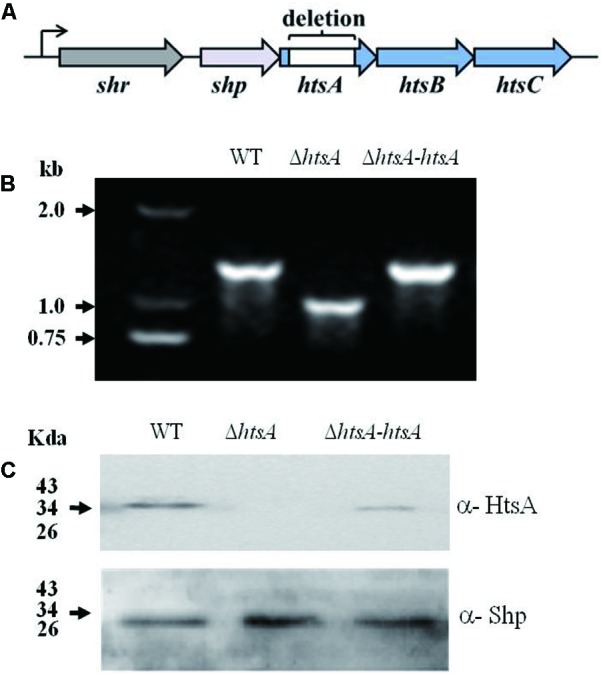
Construction of a Δ*htsA* mutant and Δ*htsA-htsA* revertant. **(A)** The arrangement of the *shr, shp*, and *htsABC* genes in the operon. **(B)** PCR analysis of fragments amplified from chromosomes of GAS strains. The PCR fragment detected in the Δ*htsA* mutant was 303-bp smaller than that in the wild type, and the PCR fragment detected in the Δ*htsA-htsA* revertant was identical to that in wild type. **(C)** Western blotting analysis of GAS strains. HtsA protein was expressed in wild type and Δ*htsA*-*htsA* strains but not in the Δ*htsA* mutant. Shp was used as a loading control.

The arrangement of the *shr*, *shp*, and *htsABC* genes in the operon was showed in **Figure 1A**. To assess the role of HtsA in GAS pathogenesis, an Δ*htsA* was constructed. To rule out the possibility that a second mutation was introduced during the construction of the mutant, a revertant of Δ*htsA*, named Δ*htsA-htsA*, was constructed using the same strategy by introducing the wild type full-length *htsA* gene into Δ*htsA*. The mutation and complementation of *htsA* were confirmed by PCR and western blotting. As expected, the PCR fragment detected in the mutant was smaller than that of the wild type. The mutant lacked the desired 303-bp fragment that encodes amino acids Asp152 to Phe252 of *htsA*. The PCR fragment of the revertant was identical to that of the wild type (**Figure 1B**). Furthermore, western blotting analysis detected HtsA protein expression in the wild type and the revertant but not in the mutant, further confirming that *htsA* was successfully deleted. As a loading control, Shp expression was also detected, and Shp was expressed in all three strains, including wild type, Δ*htsA*, and Δ*htsA-htsA* (**Figure 1C**).

### Deletion of *htsA* Attenuated GAS Virulence in Mice

**FIGURE 2:**
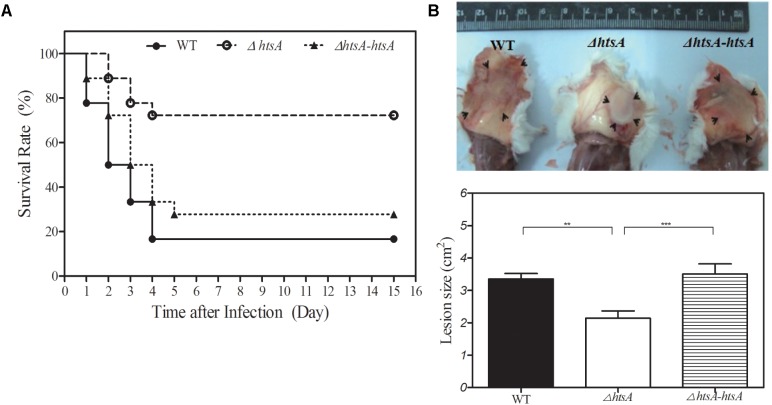
Deletion of *htsA* attenuated GAS virulence in mice. **(A)** Kaplan–Meier survival curves showing the relative rates of mouse mortality caused by wild type, Δ*htsA* and Δ*htsA-htsA* strains. Groups of mice (*n* = 18) were injected subcutaneously with ∼2.0 × 10^8^ CFU of GAS strains, and survival rates were observed daily for 15 days. Compared to wild type and Δ*htsA-htsA*, the survival rate in mice infected with Δ*htsA* was significantly higher. **(B)** Skin lesion sizes caused by wild type, Δ*htsA* and Δ*htsA-htsA* strains. Groups of mice (*n* = 7) were injected subcutaneously with ∼2.0 × 10^8^ CFU of GAS strains, and skin lesion sizes were calculated by measuring the length and width at the longest point of the lesion (length by width) at 24 h postinoculation. Compared to wild type and Δ*htsA-htsA*, the average area of skin lesion sizes in mice infected with Δ*htsA* was significantly smaller. ^∗∗^*P* < 0.01, ^∗∗∗^*P* < 0.001.

To determine the role of HtsA in GAS virulence, a mouse model of subcutaneous infection was used in this study. Groups of mice were infected subcutaneously with wild type, Δ*htsA*, or Δ*htsA-htsA* strain, and survival rates were determined daily for 15 days (**Figure 2A**). At a dose of ∼2.0 × 10^8^ CFU, 75% of the mice infected with Δ*htsA* survived until the end of 15 days, while 15% of the mice infected with wild type survived. The difference in the survival rate between the wild type and Δ*htsA* was statistically significant (*P* < 0.001), indicating that deletion of the *htsA* gene attenuates GAS virulence in the mouse model of subcutaneous infection. In addition, only 25% of the mice infected with Δ*htsA-htsA* survived, indicating that the revertant strain restored GAS virulence (Δ*htsA-htsA* versus Δ*htsA*, *P* < 0.001; Δ*htsA-htsA* versus wild type, *P* > 0.05).

To further characterize the effect of deletion of *htsA* on subcutaneous infection, skin lesion sizes of mice were measured at 24 h postinoculation (**Figure 2B**). The average area of skin lesions in mice infected with Δ*htsA* was 2.14 ± 0.22 cm^2^, which was significantly smaller than that observed in mice infected with wild type (3.35 ± 0.17 cm^2^, *P* < 0.01) or Δ*htsA-htsA* (3.51 ± 0.31 cm^2^, *P* < 0.001); however, there was no significant difference in skin lesion size between wild type and Δ*htsA-htsA* (*P* > 0.05). Thus, these results further confirmed that the *htsA* gene is critical for GAS virulence in a mouse model of subcutaneous infection.

### Deletion of *htsA* Reduced GAS Systemic Dissemination in Mice

**FIGURE 3:**
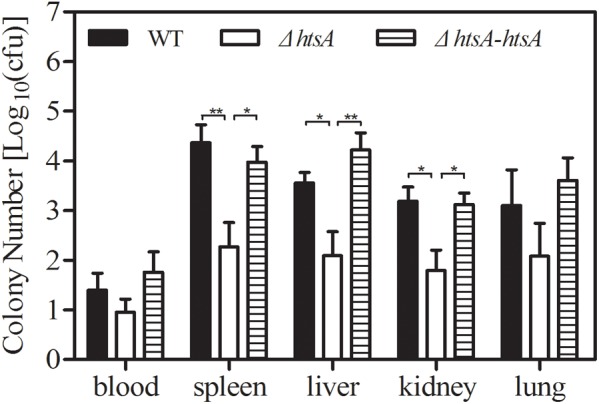
Deletion of *htsA* reduced GAS systemic dissemination in mice. Groups of mice (*n* = 7) were infected with ∼2 × 10^8^ CFU of GAS strains, and then the GAS load in heparinized blood and tissue homogenates from spleen, liver, kidneys, and lungs was determined at 24 h postinoculation. The GAS load in samples was expressed as log_10_ CFU. GAS log_10_ CFU values in the spleen, liver, and kidneys of mice infected with Δ*htsA* were significantly lower than those observed in mice infected with wild type, and Δ*htsA-htsA* could restore the virulence of GAS. ^∗^*P* < 0.05, ^∗∗^*P* < 0.01.

To determine whether HtsA is required for systemic dissemination of GAS, groups of mice were injected with ∼2 × 10^8^ CFU of GAS strains; at 24 h postinfection, heparinized blood and tissue homogenates from spleen, liver, kidneys, and lungs were plated on THY agar plates for quantification of bacterial load. The GAS load in samples was expressed as log_10_CFU. As shown in **Figure 3**, GAS log_10_CFU values in the spleen, liver, and kidney of mice infected with Δ*htsA* were 4.37 ± 0.34/100 mg, 3.56 ± 0.21/100 mg, and 3.19 ± 0.29/100 mg, which were significantly lower than those observed in mice infected with wild type (spleen, *P* < 0.01; liver, *P* < 0.05; kidney, *P* < 0.05). Although no significant differences were detected (*P* > 0.05), GAS CFU in the blood and lungs of mice infected with Δ*htsA* decreased compared to mice infected with wild type. GAS CFU values detected in different samples of mice infected with Δ*htsA-htsA* were similar to those in wild type, indicating that Δ*htsA-htsA* could restore the virulence of GAS. These results suggest that HtsA is required for systemic dissemination of GAS in a mouse model of subcutaneous infection.

### Deletion of *htsA* Impeded GAS Adhesion to and Invasion of Human Laryngeal Epithelial (Hep-2) Cells

**FIGURE 4:**
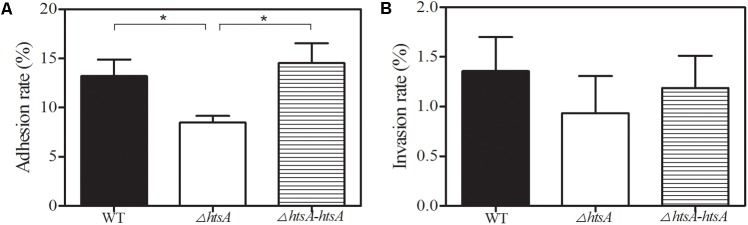
Deletion of *htsA* impeded GAS adhesion to and invasion into human laryngeal epithelial (Hep-2) cells. **(A)** GAS adhesion to Hep-2 cells. Adhesion of GAS strains to endothelial cells was assayed after 2 h of incubation. The data shown represent the percentages of adherent cells in relation to the initial bacterial inoculum. The mean adhesion rate to Hep-2 cells for Δ*htsA* was significantly lower than that for wild type and Δ*htsA-htsA*. **(B)** GAS invasion into Hep-2 cells. Invasion of GAS strains into endothelial cells was determined by an antibiotic protection assay. The data shown represent the percentages of invasive cells in relation to the initial bacterial inoculum. There was a tendency toward a reduced invasion rate in Δ*htsA* compared to that in wild type and Δ*htsA-htsA*. ^∗^*P* < 0.05.

Adhesion to and invasion of host epithelium cells is the key first step in GAS infection. To assess the role of HtsA in GAS adherence to and invasion of host cells, GAS strains were incubated with Hep-2 cells. Adherence of GAS strains to epithelial cells was assayed after a 2-h incubation, and invasion of GAS strains into epithelial cells was determined by an antibiotic protection assay. The result showed that the mean adhesion rate of Δ*htsA* to Hep-2 cells was 8.5%, which was significantly lower than that of wild type (13.2%, *P* < 0.05), and the Δ*htsA-htsA* revertant restored the wild type phenotype, resulting in a similar level of adhesion (14.5%) (**Figure 4**). Although no significant differences were found (*P* > 0.05), there was a tendency toward a reduced invasion rate in Δ*htsA* (0.9%) compared to wild type (1.4%) and Δ*htsA-htsA* (1.2%). Taken together, the above results suggest that HtsA might play a role in GAS adhesion and invasion of host epithelial cells.

### Deletion of *htsA* Decreased GAS Resistance to Phagocytosis

**FIGURE 5:**
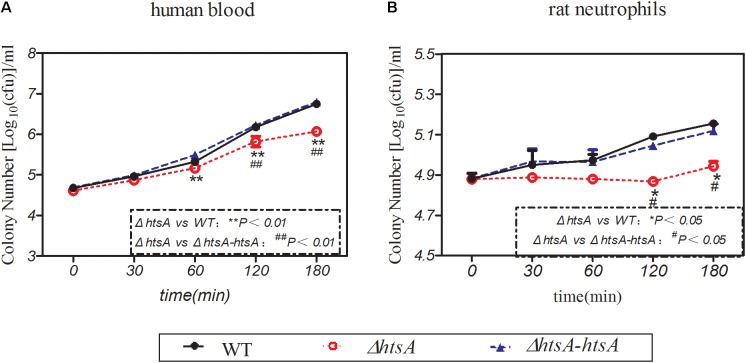
Deletion of *htsA* decreased GAS resistance to phagocytosis. **(A)** Deletion of *htsA* decreased survival in blood. Wild type, Δ*htsA*, and Δ*htsA-htsA* strains were incubated with heparinized blood, and bacterial CFU were determined at 0 min, 30 min, 60 min, 120 min, and 180 min postincubation. After 60 min of incubation, the growth of Δ*htsA* in human blood was significantly slower than that of wild type and Δ*htsA-htsA*; **(B)** deletion of *htsA* decreased survival in rat neutrophils. Wild type, Δ*htsA*, and Δ*htsA-htsA* strains were incubated with purified rat neutrophils, and bacterial CFU values were determined at 0 min, 30 min, 60 min, 120 min, and 180 min postincubation. At 120 min and 180 min of incubation, the growth of Δ*htsA* in rat neutrophils was significantly slower than that of wild type and Δ*htsA-htsA*.

GAS resistance to phagocytosis was investigated by observing GAS survival in whole human blood or in purified rat neutrophils. To investigate the survival of GAS in whole human blood, wild type, Δ*htsA*, and Δ*htsA-htsA* strains were incubated with heparinized blood, and bacterial CFU values were determined at 0 min, 30 min, 60 min, 120 min, and 180 min postincubation. **Figure 5A** shows the survival of three GAS strains in human blood at different time points. In the first 30 min of incubation, the three GAS strains grew at similar rates. After 60 min of incubation, the survival of Δ*htsA* in human blood was significantly lower than that of the wild type (*P <* 0.01) or Δ*htsA-htsA* (*P <* 0.01), and there was no significant difference between the Δ*htsA-htsA* and wild type (*P >* 0.05). This result indicated that the deletion of *htsA* could lead to decreased survival in human blood. To further investigate the role of HtsA in the resistance of GAS to phagocytosis, wild type, Δ*htsA*, and Δ*htsA-htsA* strains were incubated with purified rat neutrophils, and bacterial CFU were determined at 0 min, 30 min, 60 min, 120 min, and 180 min postincubation. **Figure 5B** shows that survival of Δ*htsA* in rat neutrophils was significantly lower than that of wild type (*P <* 0.05) or Δ*htsA-htsA* (*P <* 0.05) at 120 min and 180 min postincubation. Together these data support the idea that the reduced virulence of Δ*htsA* is associated, at least in part, with decreased resistance of GAS to phagocytosis.

### Deletion of *htsA* Induced Higher Neutrophil Recruitment at the Skin Infection Site of Mice

**FIGURE 6:**
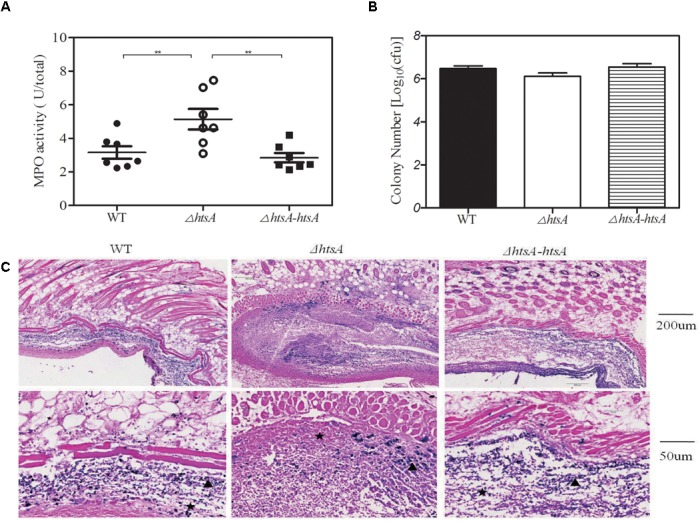
Deletion of *htsA* induced higher neutrophil recruitment at skin infection sites of mice. Groups of mice were subcutaneously injected with wild type, Δ*htsA*, or Δ*htsA-htsA* strains at a dose of ∼2.0 × 10^8^ CFU, and the skin containing the infection area was excised and homogenized at 24 h postinfection. **(A)** MPO activity at skin infection sites of mice. The MPO activity (U/total) of mice infected with Δ*htsA* was higher than that of mice infected with wild type or Δ*htsA-htsA*. **(B)** The total bacterial CFU at skin infection sites of mice. There were no statistically significant differences in total bacterial CFU among the three GAS strains. **(C)** Microscopic pictures of Gram staining at a skin infection site. 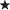
inflammatory cells, 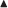
bacteria. ^∗∗^*P* < 0.01.

Myeloperoxidase (MPO) is a key inflammatory peroxidase enzyme secreted by activated neutrophils and macrophages. Thus, neutrophil recruitment at the infection site of skin was estimated by determining MPO activity. Groups of mice were subcutaneously injected with wild type, Δ*htsA*, and Δ*htsA-htsA* strains at a dose of ∼2.0 × 10^8^ CFU, and then the skin containing the infection area was excised and homogenized at 24 h postinfection. Meanwhile, the homogenates were serially diluted and cultured on THY plates overnight to calculate the CFU. As shown in **Figure 6**, the MPO activity (U/total) in mice infected with Δ*htsA* was 5.140 ± 0.610, 1.6-fold higher than that in mice infected with wild type (3.161 ± 0.372, *P* < 0.01) and 1.8-fold higher than that in mice infected with Δ*htsA-htsA* (2.849 ± 0.281, *P* < 0.01). We did not detect statistically significant differences in total bacteria CFU in skin among the three GAS strains (**Figure 6B**), indicating that the enhanced neutrophil recruitment at skin infection site did not kill Δ*htsA.* However, Gram staining indicates that Δ*htsA* bacteria site was surrounded by an intense layer of inflammatory cells (pink), and Δ*htsA* bacteria were associated with intense necrotic materials (pink stain inside infection sites). In contrast, wild type and Δ*htsA*-*htsA* bacteria (blue) were largely free of necrotic cells and invaded skin to cause larger lesions than Δ*htsA* bacteria (**Figure 6C**). These pathological features suggest that the enhanced neutrophil recruitment at Δ*htsA* sites prevents GAS skin invasion and systemic dissemination, thereby attenuating the virulence.

### Deletion of *htsA* Induced High Production of Cytokines in Mice

**FIGURE 7:**
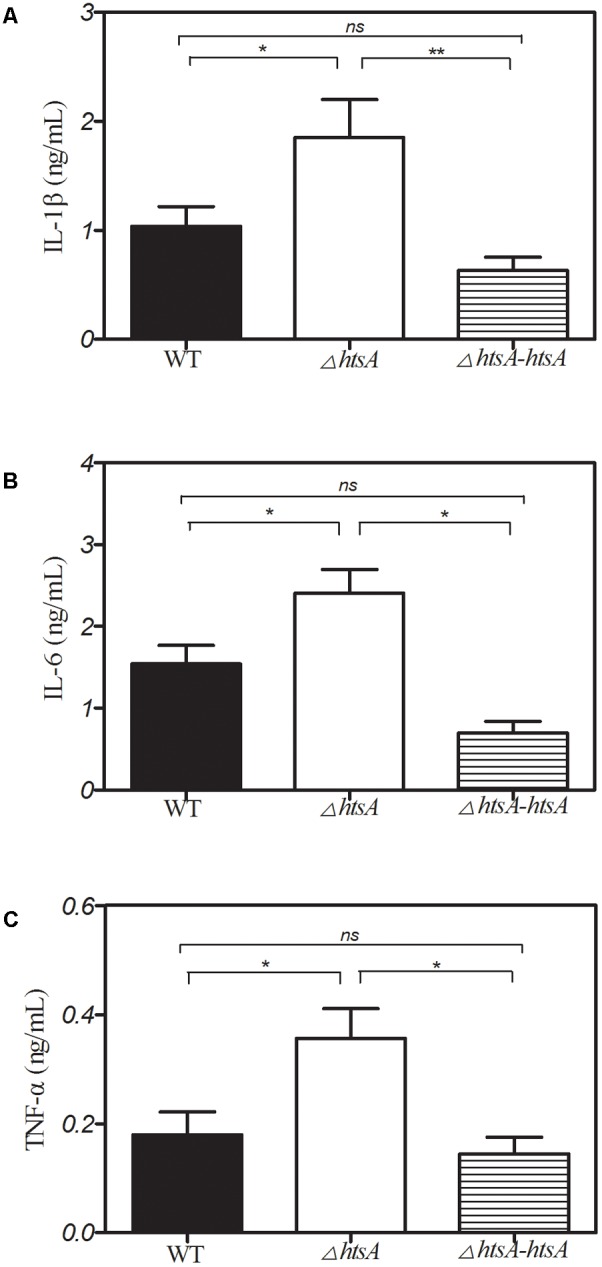
Deletion of *htsA* induced high production of cytokines in mice. Groups of mice (*n* = 7) were subcutaneously injected with 2 ml of air to form an air pouch and inoculated with ∼2 × 10^8^ CFU of bacterial strains. At 24 h postinfection, air pouch exudate was collected, and the cytokine levels of IL-1β, IL-6, and TNF-α in the exudate were determined by ELISA assay. The result showed that cytokine levels of IL-1β, IL-6, and TNF-α in the mice infected with Δ*htsA* were higher than those in mice infected with wild type or Δ*htsA-htsA*. ^∗^*P* < 0.05, ^∗∗^*P* < 0.01.

Cytokines are important for recruiting phagocytic cells to local infection sites. Thus, we hypothesized that deletion of *htsA* may enhance neutrophil recruitment by increasing the production of cytokines. To test this, a mouse air pouch model of infection was used. Mice were subcutaneously injected with 2 ml of air to form an air pouch and then inoculated with 0.2 ml of ∼2 × 10^8^ CFU of bacterial strains into the skin air pouch. At 24 h postinfection, the exudate isolated from each air pouch was subjected to ELISA to detect the levels of interleukin (IL)-1β, IL-6, and tumor necrosis factor alpha (TNF-α). The results are shown in **Figure 7**, where cytokine levels of IL-1β, IL-6, and TNF-α in mice infected with Δ*htsA* were higher than those in mice infected with wild type (IL-1β, *P* < 0.05; IL-6, *P* < 0.05; TNF-α, *P* < 0.05) or Δ*htsA-htsA* (IL-1β, *P* < 0.01; IL-6, *P* < 0.05; TNF-α, *P* < 0.05). There were no significant difference in levels of IL-1β, IL-6, and TNF-α between wild type and Δ*htsA*-*htsA* infections (*P >* 0.05).These results support our hypothesis that deletion of *htsA* could induce high production of cytokines at local infection sites in mice, resulting in greater neutrophil recruitment.

## Discussion

The growth of GAS also requires iron, and heme is an important source of iron for GAS. The GAS heme acquisition machinery contains Shr, Shp, and HtsABC. HtsA, the lipoprotein component of HtsABC, binds heme and directly assimilates heme from Shp (Lei et al., 2003).

Previous studies did not study the relationship between HtsA and virulence and its mechanism. The major finding in this study is that HtsA is important for GAS virulence. We found that deletion of the *htsA* gene increases the survival rate, decreases skin lesion size, and decreases GAS systemic dissemination in a mouse model of subcutaneous infection. These results demonstrated that the *htsA* gene is critical for full virulence in a mouse model of subcutaneous GAS infection and suggest that HtsA may be a new virulence factor in addition to being a component of the HtsABC transporter of GAS for heme acquisition. Our unpublished pilot microarray and real-time PCR analysis of gene transcription showed that the expression of other virulence factors including *emm*, *sic*, *slo*, *covS*, *hasA*, *sdaD2*, *ska*, *speB*, and *rgg* was not notably altered after *htsA* deletion, which also supports our assumption that HtsA may be a new virulence factor. In addition, the gene transcription results detected by real-time PCR showed that the relative expression levels of *shr*, *shp*, *htsB*, and *htsC* were similar in wild type strain and Δ*htsA* mutant strain, supporting that the deletion of *htsA* did not cause the polar effects (Supplementary Figure S1).

Fe acquisition systems are virulence factors in many bacteria (Griffiths, 1991). Inactivation of iron-acquisition system-encoding genes correlates with virulence attenuation of many bacterial pathogens in animal models (Sritharan, 2000). In GAS, an early report by Fisher et al. (2008) found that the *shr* mutation reduced the virulence of zebrafish infection. Other reports by Dahesh et al. (2012) and Eichenbaum (2012) found that Shr is required for full virulence of serotype M1 GAS in mouse models of invasive disease. Our recent study (Zhang et al., 2017) found that Shp significantly contributes to GAS skin invasion, systemic infection, and virulence. Our results in this study further confirm and enrich the hypothesis that heme uptake proteins are related to bacterial virulence. Previous studies were all on bacterial surface proteins, and HtsA is a transporter component in the cell wall; our study also found that HtsA has a certain degree of immune protection (data not shown), so future infection treatment is a good goal. GAS genomes encode three transporters that can take up iron: MtsABC, FtsABCD, and HtsABC. Only the HtsABC transporter binds and acquires heme, while the other two transporters, MtsABC and FtsABCD, are involved in the acquisition of Fe^3+^ and Mn^2+^ and Fe^3+^ ferrichrome, respectively (Janulczyk et al., 2003; Hanks et al., 2005). A previous study demonstrated that MtsABC is important for GAS virulence (Janulczyk et al., 2003). Because free Fe^3+^ is not available in humans due to its extremely low solubility under physiological conditions, the attenuation of GAS virulence by MtsA inactivation may not be attributable to the function of the transporter in Fe^3+^ acquisition. This implies that MtsABC may not be involved in iron acquisition *in vivo* because free Fe^3+^ is not available in mammalian hosts; thus, the roles of MtsABC in iron acquisition and virulence are less important in vivo. FtsABCD likely borrows siderophores from other bacteria at non-invasive infection sites such as the tonsils in acute streptococcal pharyngitis. FtsABCD would hardly be able to be involved in iron acquisition in invasive infections without endogenous and exogenous siderophores. Since FtsABCD is not critical to GAS virulence and MtsABC may not be involved in iron acquisition *in vivo*, the HtsABC transporter could be a more important iron transporter during invasive GAS infections.

Colonization of the host epithelium is a key first step in GAS infection, and cell surface proteins such as M protein and fibronectin-binding proteins are important for this process (Brouwer et al., 2016). Nobbs et al. (2009) summarized that metal ion transporters have been implicated in streptococcal colonization processes and may influence adherence and biofilm formation as a putative adhesins. Fisher et al. (2008) found that Shr can function as an adhesion, and the inactivation of *shr* resulted in a 40% reduction in attachment to human epithelial cells in comparison to the parent strain. Although Dahesh et al. (2012) reported that Shr does not mediate binding to Hep-2 cells, they found that mutation of *shr* resulted in a significant reduction in laminin binding and a tendency toward reduced fibronectin binding. In this study, we investigated the effect of *htsA* deletion on the adherence to and invasion of human epithelial cells *in vitro*. We found that *htsA* deletion decreased GAS adherence to Hep-2 cells *in vitro*, suggesting that HtsA participates in or assists other virulence factors with adhesion to epithelial cells. Our study demonstrated that HtsA enhanced GAS virulence in both subcutaneous infection models *in vivo* and epithelial cell models *in vitro*.

During a bacterial infection, innate and adaptive host immune responses are fundamental for defense against infection (Fieber and Kovarik, 2014). Neutrophils are the most prominent cellular component of innate immunity, and they are essential for host defense against bacterial infections (Lu et al., 2014). In this experiment, we found that the Δ*htsA* mutant strain had significantly decreased survival in human blood and rat neutrophils compared to the wild type strain, indicating that deletion of *hts*A could lead to decreased bacterial resistance to neutrophils *in vitro*. In addition, we detected neutrophil recruitment to infected skin sites by detecting MPO activity and found that deletion of *htsA* could cause increased MPO activity at infected skin sites, indicating that more neutrophils were recruited after infecting with the Δ*htsA* mutant strain *in vivo*. The MPO activity indirectly reflects the level of the neutrophils *in vivo*. These results further confirmed the idea that the reduced virulence of ΔHtsA is associated, at least in part, with decreased resistance to phagocytosis.

Cytokines play key roles in protecting the host against bacterial infection by regulating the innate immune response (Soderholm et al., 2018). For example, a *Streptococcus* ADP-ribosyltransferase, SpyA, triggers an IL-1β-dependent innate immune response pathway that is critical in defense against invasive bacterial infection (Lin et al., 2015). Cytokines are important for recruiting phagocytic cells to infection sites and killing the invading bacteria (Mohammadi et al., 2016). On the other hand, some cytokines could directly inhibit and eliminate bacteria during microbial infection (Delves and Roitt, 2000). Our data in this study showed that deletion of *htsA* resulted in a significant increase in the levels of TNF-α, IL-1β, and IL-6 in the air pouch exudates of mice. Based on our result and previous reports, we hypothesize that deletion of *htsA* may promote the secretion of cytokines and enhance phagocytic cell recruitment during GAS infection. We also found that the Δ*htsA* strain growth curves were almost the same as those of the wild type strain *in vitro*, but the competitive ratio was lower *in vivo* (Supplementary Figure S2), which may also be related to the host early innate immune response.

The present study provides the first direct evidence that HtsA is important to GAS virulence in a mouse model of subcutaneous GAS infection, and it will advance our understanding of GAS pathogenesis. The mechanism between bacterial iron metabolism and early immune response needs further study.

## Author Contributions

YS, XZ, MC, CL, YZ, and DW conducted the experiments. YS and HZ designed the study and wrote the manuscript. All authors gave intellectual input to the study and approved the final version of the manuscript.

## Conflict of Interest Statement

The authors declare that the research was conducted in the absence of any commercial or financial relationships that could be construed as a potential conflict of interest.
