# Supplementary material for: The Heme Transporter HtsABC of Group A Streptococcus Contributes to Virulence and Innate Immune Evasion in Murine Skin Infections
Source: Front Microbiol. 2018 May 25;9:1105. doi: 10.3389/fmicb.2018.01105 (PMC5981463; doi:10.3389/fmicb.2018.01105)
Supplement: Supplementary file 1 [file Data_Sheet_1.DOCX]

Supplementary Material

Article Title：The Heme Transporter HtsABC of Group A *Streptococcus* Contributes to Virulence and Innate Immune Evasion in Murine Skin Infections

Yingli Song, Xiaolan Zhang, Minghui Cai, Chunmei Lv, Yuan Zhao, Deqing Wei, Hui Zhu*

*** Correspondence:** Corresponding Author: dzhuhui@aliyun.com.

## Supplementary Figures


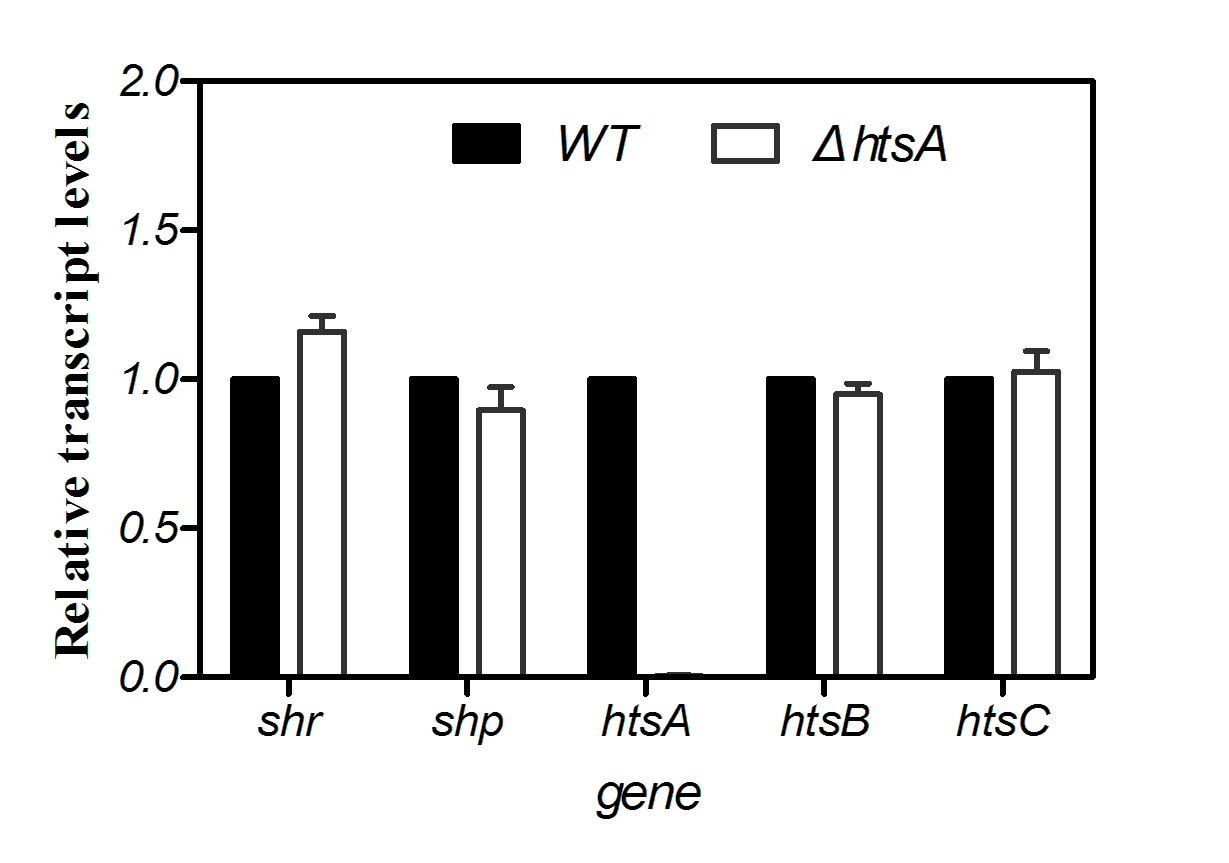


**Supplementary Figure 1.** The The effect of the deletion of *htsA* on the relative expression levels of *shr*, *shp*, *htsB*, and *htsC*. RNA samples were isolated from wild type and *htsA* mutant strain, and relative expressions of genes were detected by real-time PCR. gyrA was as the reference gene.


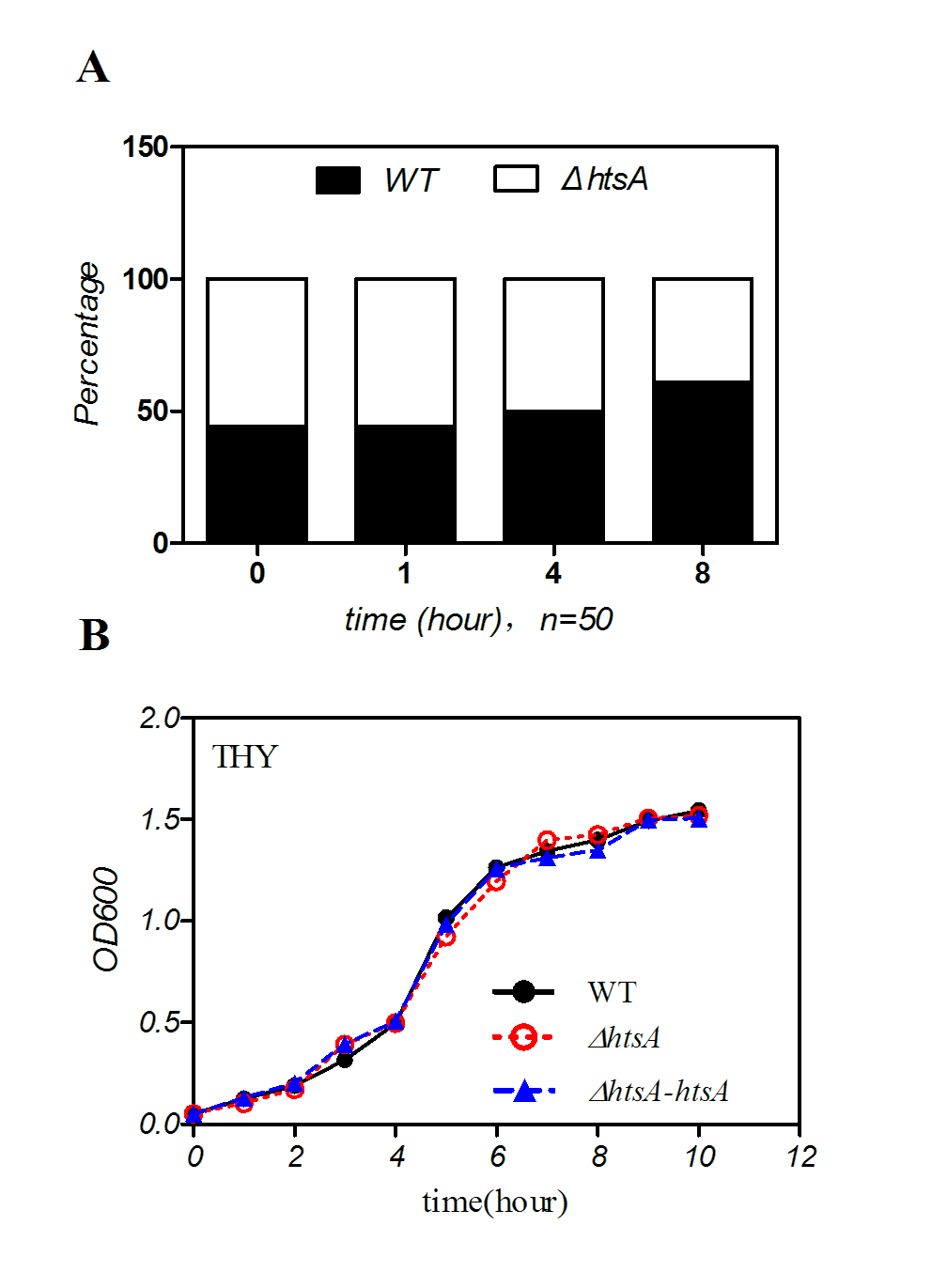


**Supplementary Figure 2.** The effect of the deletion of *htsA* on GAS growth. (A) Competition growth of wild type and the *htsA* mutant at a subcutaneous infection site in vivo. Mice were administered an equivalent amount of the wild type and *htsA* mutant strain by subcutaneous injection. At 0h, 1h, 4h and 8h postinoculation, subcutaneous tissue homogenate at appropriate dilutions was plated in THY, and colony PCR was used to determine the percentage of the Δ*htsA* to the wild type strain in the subcutaneous infection site (n=50). (B) Growth of wild type and the *htsA* mutant in normal THY medium in vitro. The growth curves were monitored by measuring the optical density at 600 nm for 10h from a starting OD600 of 0.05. Data were pooled and normalized from two independent experiments, each performed in triplicate.
